# Load-Bearing Capacity of Beams Reinforced with Composite Rebar in Regard to Existing Guidelines

**DOI:** 10.3390/ma14206116

**Published:** 2021-10-15

**Authors:** Norbert Olczyk, Jarosław Błyszko, Mateusz Techman

**Affiliations:** Faculty of Civil and Environmental Engineering, West Pomeranian University of Technology, Szczecin, al. Piastow 50a, 71-311 Szczecin, Poland; norbert.olczyk@zut.edu.pl (N.O.); jaroslaw.blyszko@zut.edu.pl (J.B.)

**Keywords:** GFRP bars, concrete reinforcement, design standards, failure mechanism

## Abstract

Non-metallic reinforcement such as fiber-reinforced polymer (FRP) is now being increasingly used in construction. Despite numerous similarities, elements reinforced with non-metallic bars work differently from the ones reinforced with steel bars, including cracking and failure mode. The examination of the stress state in these elements, so important for their proper design, raises many difficulties. The article presents the results of tests of bended beams reinforced with GFRP bars. The results of the experimental tests were compared with calculations based on selected design instructions. The results have shown that beams reinforced with GFRP exhibit increased cracking, higher deflection, and often mode of failure through crushing of concrete. The results have shown that in bended elements reinforced with the GFRP bars, the rebar often does not achieve the strength declared by the manufacturer. The study has shown that theoretical values of load-bearing capacity of beams reinforced with composite rebar differ greatly between different guidelines and instruction. The analysis showed that the use of GFRP bars as a replacement for steel bars is possible in demanding environmental conditions. However, excessive deflections and cracks may result in limited application due to overall serviceability requirements of the element.

## 1. Introduction

Concrete elements with steel bar reinforcement are widely used and well-recognised. Over the course of a century, there have been many articles on the theory of their operation and various studies that verified those assumptions. Reinforcing steel, regardless of its strength, has basically unchanged deformability parameters, despite a significant increase in the quality of reinforcing steel over the past decades. The calculations of the cracking moment and deflections of the steel-reinforced beam are given in many standards and the literature.

New buildings have high requirements, particularly for durability and load-bearing capacity. One of the solutions is to use an alternative to steel reinforcement—composite bars [1]. The use of FRP (fiber-reinforced polymer) bars is continuously increasing [2,3]. The most common types of non-metallic reinforcement are made out of carbon (CFRP), aramid (AFRP), glass (GFRP), and basalt (BFRP). Low weight, high tensile strength [4,5], and very high corrosion resistance allow the use of FRP composites when erecting concrete buildings exposed to an aggressive environment. Such objects/elements may include breakwaters, foundations, seashore facilities, or sewage treatment plants [6].

The non-metallic reinforcement has a high strength but about 3–4 times lower modulus of elasticity than steel. All fibers behave linearly elastic during tension [7,8]. The carbon and aramid fibers are anisotropic. They have different mechanical and thermal properties in the main directions, whereas glass and basalt fibers can be considered as an isotropic material [9,10]. The composite reinforcement is not limited to bars but also can be made into mesh, mats, or ropes. Reinforcement may have, depending on the need, a round or square cross-section, hollowed or full [5].

Despite certain similarities of composite bars to steel, the use of this type of reinforcement can lead to unusual phenomena in reinforced concrete structures. A steel-reinforced structure behaves differently under constant load than a structure reinforced with FRP bars.

The problem of measuring the material strain of GFRP reinforced beams is due to the relatively small compression zone. Assuming the limiting height of the compression zone according to Equation (1), we obtain its height as 10–15% of the effective height of the beam.

(1) $$\xi =\frac{x}{d}=\frac{\varepsilon_{\mathrm{cu}}}{\varepsilon_{\mathrm{cu}}+\varepsilon_{s}}$$

where ε_cu_—limit strain of concrete, ε_s_—limit strain of steel, d—construction height, and x—height of compression zone

For example, for concrete class C50/60 and bars with strength f_u_ = 1000 MPa and Young modulus E = 50,000 MPa, we obtain:

(2) $$\frac{3.5}{3.5+20}=0.149\approx 15\%\cdot d$$

Therefore, the compression zone in the beam at the ultimate limit state occupies a small (a few centimetres) height. Another consequence of GFRP reinforced elements, which is directly related to the above relationship, is the deep cracking. The high strength of GFRP bars leads to a reduction of the reinforcement area. Therefore, the stresses in the bars are already high when the first crack occurs, especially in low-reinforced elements. High stress means high deformation of the reinforcement and a wide and deep crack. The consequence of this is a higher deflection of elements reinforced with composite bars.

Testing the stress state of these elements, so important for their proper design, raises many difficulties within the technical and interpretative aspects. Many studies can be found that focus on determination of the properties and application of fiber-reinforced polymer (FRP) bars [11,12,13,14].

Studies conducted by Pawłowski [15] regarded short-term ultimate load capacity and deflection of bended elements reinforced with BFRP bars. Freely supported slabs were tested in the four-point bending test. The slabs had different degree of reinforcement and concrete strength. The results allowed the formulation of a number of conclusions, proving that bended concrete elements reinforced with BFRP bars exposed to short-term load can be designed using typical analytical models and norms used for element reinforced with other types of non-metallic reinforcement.

Typically, composite materials are used as an external strengthening method for reinforced concrete elements. Products such as composite meshes, grits, tapes, and other types of reinforcement can be installed in the tension zone of the element to enhance its strength [16,17]. However, only very limited number of studies takes on the topic of FRP bars in view of available standards for internally reinforced elements [1,18,19].

The design methods and instructions for designing concrete structures reinforced with non-metallic bars can be found in standards and reports from Japan [20], Canada [21,22], United States [23], and Europe [24], Fib Bulletin-2007 [7], and Eurocode 2 Appendix [25].

Designing of structures reinforced with the FRP bars is based on the methods for typical reinforced concrete structures for both the serviceability limit state (SLS) and ultimate limit state (ULS) [26,27]. The differences can be found in the linearly elastic operation of the FRP bars and no plastic reserves, which is included by additional material and calculation factors [28].

The article presents the results of tests of bended beams reinforced with GFRP bars, emphasising the difficulties that arise during measurements at the test stand. The results of the experimental tests were compared with calculations based on selected design instructions, highlighting the differences.

## 2. Materials and Methods

### 2.1. Specimen

**Figure 1:**
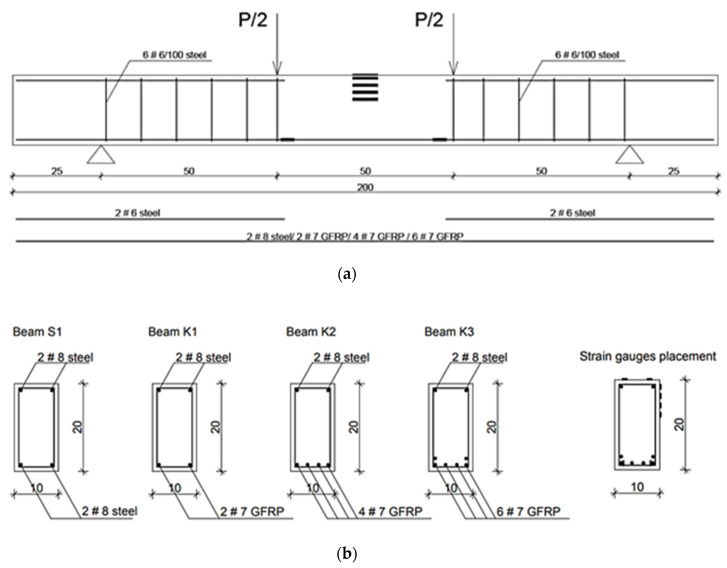
(**a**) Beam longitudal section; (**b**) The cross-section of studied beams.

The study was performed on four full-scale reinforced concrete beams. The beams were casted with a high-performance concrete and had the dimensions of 100 × 200 × 2000 mm. The proportions of the dimensions (h/b = 2; l/h = 10) are typical for reinforced concrete building elements. Real-life bended elements are, however, two or three times larger. The beam differed with the type of reinforcement in the tension zone. This assumption of this study was that the beams were designed for two different types of failure: either by exceeding the strength of reinforcement, as in the case of beams S1 (steel yielding) and K1 (breaking the GFRP bars), or concrete crushing of compression zone, as in beams K2 and K3. The schematic of beams’ reinforcement is visible in Figure 1a,b. The composite rebar was chosen from available sizes to be similar to the steel bars. Beams K3 and K4 had an increased degree of reinforcement due to increased number of bottom bars. The degree of reinforcement in this study varied from 0.38% to 1.15%. The bars were held together by steel stirrups. The stirrups were installed only near the support zones, not in the middle of the span. The stirrups were distributed every 10 cm. The setup was made to isolate the influence and behaviour of composite rebar, compared to traditional steel reinforcement.

### 2.2. Material Properties

The concrete used for this study was designed and prepared at Laboratory of Reinforced Concrete Structures and Concrete Technology of West Pomeranian University of Technology, Szczecin. The mix design is presented in Table 1.

The designed concrete was a self-consolidating mix, which allowed the preparation of the beams without additional mechanical compaction. In the case of GFRP bars, which are susceptible to external damage, it is important to preserve the bars in their initial state throughout the whole process of casting.

The mean compressive strength acquired in cube compression test was f_cm,cube_ = 71.7 MPa. The results allowed classifying it as a high-performance concrete C55/67. The beams were casted in a single sitting with two batches of concrete. Both batches were tested to confirm their uniformity.

The steel bars used in the study were made of a typical construction steel B500A. The properties of steel reinforcement are presented in Table 2.

**Figure 2:**
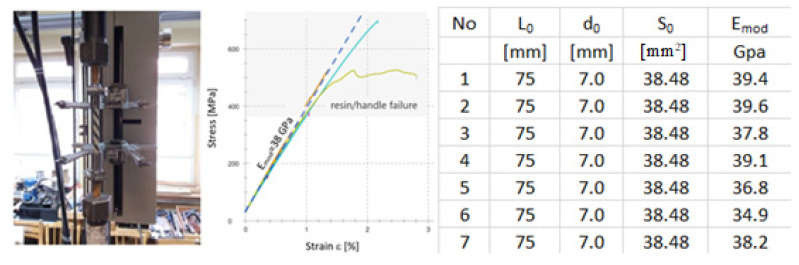
Testing of composite rebar.

The GFRP reinforcement was tested to confirm the properties given by the manufacturer. Due to test stand limitations and the necessity of fitting the ends of bars inside a special resin grip, the length of the bars was limited. This imposed additional limitations to the test procedure. For such high tensile strength of above 1000 MPa declared by the manufacturer, it was impossible to confirm its final value. For this reason, the test was performed to a stress level of approximately 600 MPa, as Figure 2 presents. The test stand is visible in Figure 2.

The results of initial tests concurred with manufacturers data, the value of modulus of elasticity was assumed as 38 GPa for this study.

### 2.3. Test Setup

**Figure 3:**
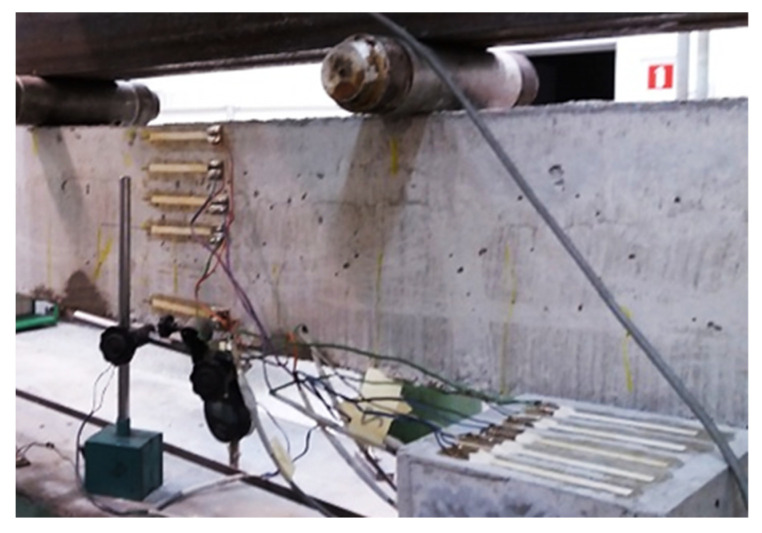
Strain gauge placement.

**Figure 4:**
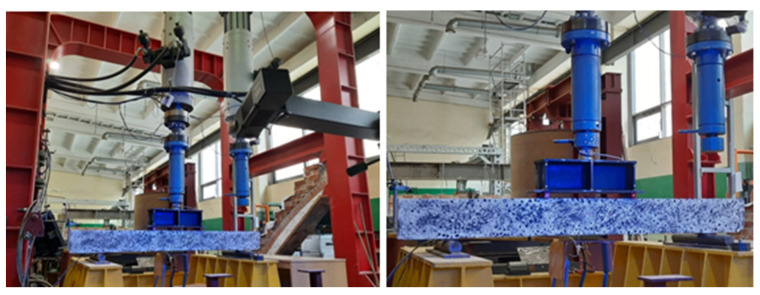
Test stand. Sample prepared for optical measurements of deformations.

Figure 3 and Figure 4 shows the test stand for samples and strain gauge placement. The spacing between the supports was 150 cm. The deflection in the middle of the span was measured and the crack propagation was registered. Deformations of the beams were measured using a set of 16 strain gauges, connected in a half-bridge system. The deformations of concrete were measured using 12 T1–T6 strain gauges (6 measuring and 6 compensating) with 75 mm base and 289 ± 1 Ω resistance, while the deformations of GFRP bars were measured using four strain gauges (two measuring and two compensating) with 20 mm base and 120 ± 1 Ω resistance.

Precise measurement of the strain for such a small compression zone is not possible. It is therefore assumed that a single strain gauge, approximately 1 cm wide will be sufficient. It should be positioned at the center of the height of the predicted compression zone, or at the top of the beam.

Strain measurements in tension zone due to occurring cracks is sometimes difficult. Cracks in the beam appear randomly and strain gauges for measuring concrete must be longer than 50 mm (usually about 75 mm). It is therefore very common for them to be damaged during the test. In the section between the cracks, the concrete in the tension zone is exposed to low strain.

A method that allows free measurement of such beams are the non-contact optical methods, in which, unfortunately, the problem is that large deformations have to occur in order to be registered. Therefore, in bended beams, due to high deformation, the cracking is clearly visible. Those methods allow the measurement of the element as a whole, not only selected sections.

To obtain a high-precision measurement, an optical deformation measurement system ARAMIS (GOM Correlate Professional 2020 software) was applied. The ARAMIS real-time measurement system analyses the deformation by means of triangulations. The recording of the test results is based on the comparison of successively taken photos, using high-resolution digital cameras, with a set frequency. The software assigns then the coordinates for each pixel in relation to set reference points. The program compares the local deformation to the reference system. For proper preparation of the test stand, reference points have been defined on non-movable parts of the stand in order to determine the global coordinate system. In addition, during the test, it was possible to carry out a parallel analysis of the impact of the bending beam on its surroundings and press components. Figure 4 shows a sample prepared for testing and the test stand.

## 3. Results

### 3.1. Crack Pattern and Deflection

In beam reinforced with steel bars (degree of reinforcement of approximately 0.5%) the cracks appeared in a in the middle in a perpendicular direction to beam’s span. With the increase in the load the crack range increased until yield point. Then, a wide and deep crack appeared in the yield zone reaching up to the compression zone, which caused the failure.

**Figure 5:**
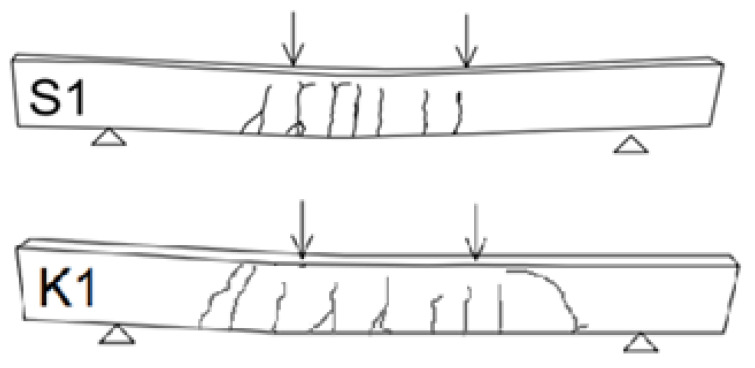

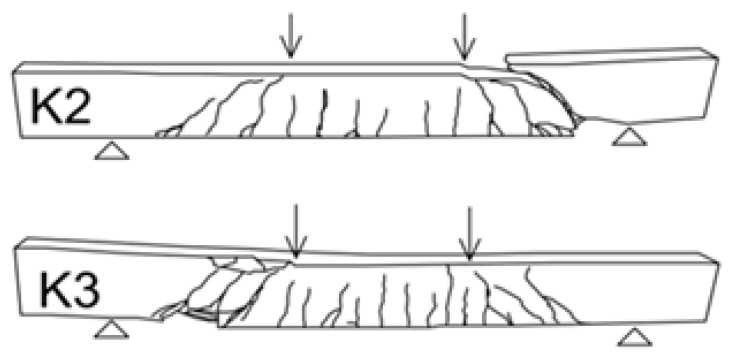
Development of cracks in studied beams at failure.

In the case of the beams reinforced with only two composite bars at the bottom (K1), the cracks development was to some point similar to the one occurring in steel reinforced member but rather larger range of cracks occurrence. The cracks from the beginning had larger width than in beams reinforced with steel bars. The cracks were perpendicular, with no diagonal ones occurring. This shows that the influence of shear forces was minimal. The span of cracks was larger than in steel-reinforced beam. The results of maximum crack width are presented in Table 3. The cracks also appeared more rapidly. Each occurrence of crack was followed by sudden increase in beams deflection. The cracks propagated almost through the whole height of the beam, leaving only small compression zone. The crack mapping is presented in Figure 5.

With the increase in the degree of reinforcement the load-bearing capacity of the beams increased. Typical perpendicular cracks appeared in the middle during the first stages of loading. Then, diagonal cracks started to appear due to shear forces within the element. The height of the compression zone was also decreased, ranging to around 30 mm, approximately 1/6 of the cross-section. In beams with higher degree of reinforcement (K2 and K3) the number of cracks was increased compared to other studied elements. The K1 beams exhibit failure due to the effect of bending forces, K2 and K3 due to shear forces. The load bearing capacity was reached when the compression zone was rapidly crushed. This caused a transfer of the centroidal axis and failure of composite rebar. Values of the maximum crack width and deflection before failure is presented in Table 3.

### 3.2. Optical Measurements

**Figure 6:**
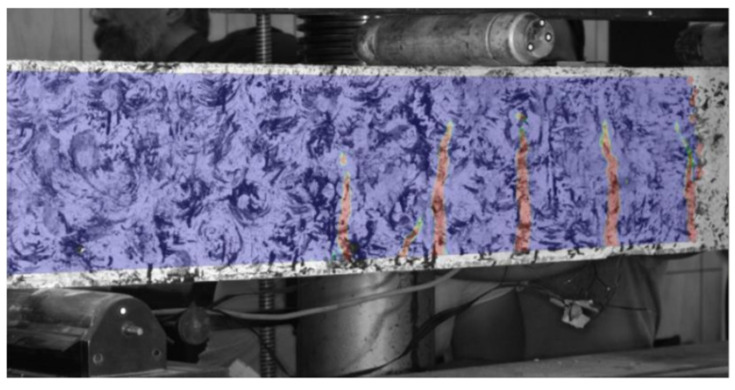
Perpendicular cracks in the element reinforced with composite bars (K2).

Optical measurement of crack development and deflections was performed for K1 and K2 beams with two and four composite bars, respectively, at the bottom. The K1 beam in most of the guidelines is designed to exhibit failure through exceeding the load-bearing capacity of the rebar. The K2 beam, with more bars, should, according to most of the guidelines, exhibit failure by exceeding stress capacity of compression zone. Failure mechanism and calculations in accordance to different guidelines is presented further in this research. The example of real-time crack measurements is shown in Figure 6. The cracks are visible as red on a blue background.

**Figure 7:**
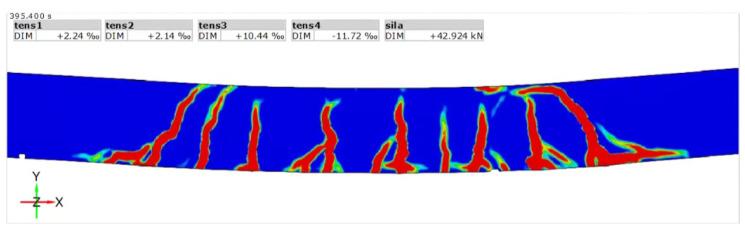
Measurement of cracks for K1 beam.

**Figure 8:**
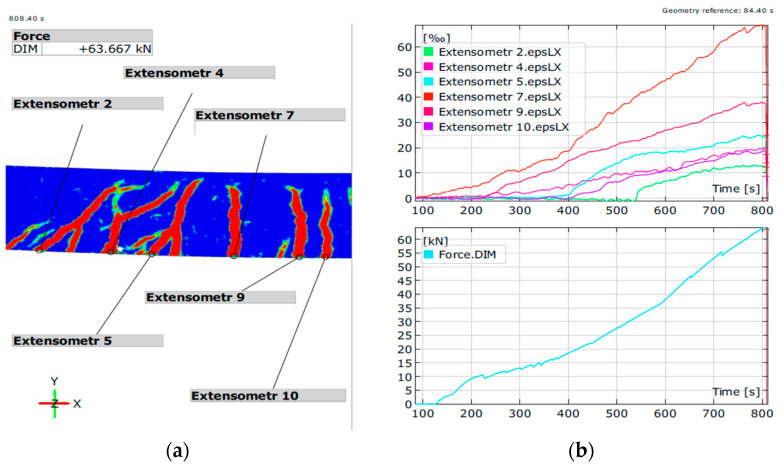
Measurement of cracks for K2 beam. (**a**) crack generation map during the test; (**b**) values of deformations (crack width) in each point at any given force.

The measuring equipment allows the application of virtual extensometers in the location of cracks, allowing for the measurement of the width. The results of the test are visible in Figure 7 and Figure 8. The values of crack width are visible as the ‰ of the base value, which in the case of this study was 20 mm, the 1/10 of the height of the beam h = 200 mm.

The results of the optical measurements concur that the cracks in beams reinforced with composite rebar are wider than typical cracks in steel-reinforced beams. The crack propagated to the compression zone before the failure. Beam K1 with only two composite rebar exhibited higher deflection. There was an increase in the number of bars; thus, the reinforcement ratio increased the stiffness of the beams.

### 3.3. Failure Mechanism and Deformations

Typical for steel reinforcement, the failure of beams was due to failure of the bars in flexural zone. For beams reinforced with increased number of composite bars, the failure was observed due to the destruction of concrete, which then caused a failure of composite bars. This highlights the importance of proper concrete works in elements reinforced with composite bars. The better the concrete works and the quality of concrete, the higher the durability of the element.

As seen in Figure 5, beams with an increased degree of reinforcement (K2 and K3) failed due to shear forces near the support zone.

**Figure 9:**
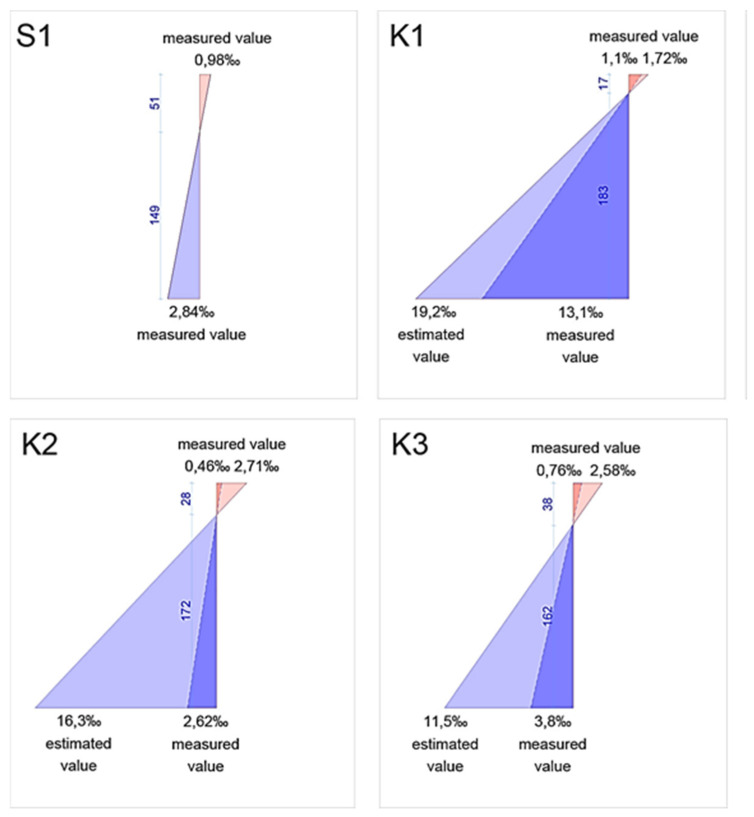
Measured and expected values of deformation in studied beams.

The deformations of the beams measured using strain gauges are presented in Figure 9.

The deformations were measured both on rebar (blue) and concrete (red). In the case of beams reinforced with composite rebar, due to the increased deformation of the bars, the strain gauges failed during the tests. The gauges installed in the compression zone registered the deformation until the failure of the beam. The presented graph for beams K1–K3 shows both last measured values (deeper shade of colour) and estimated values (lighter shade of colour) calculated based on the measurements from strain gauges installed on concrete.

Deformation of bending zone indicated yielding of the steel bar. The results of the K1 beam show extensive deformation of bars before failure. For K2 and K3 beams, the deformation in rebar does not exceed the limit values. However, the value of deformation at the top of the compression zone oscillates around the limit value for concrete deformation. With the increase in the number of bars, the stiffness of the beam increases, increasing the effective compression zone within the element. The values of deformation measured during the test concur with theoretical values presented in Table 4.

### 3.4. Comparison between the Standards

For studied beams, calculations of load-bearing capacity were conducted in accordance with four instructions. Two cases have been taken into consideration. The characteristic capacity M_rk_ was calculated as the highest possible moment, disregarding all partial factors. In the second case, the design capacity M_rd_ was calculated, including all partial factors. The values acquired in the laboratory tests (M_exp_) are given at the top of the table.

The results of the calculations for the beam reinforced with steel rebar show a good correlation between theoretical (EN 1992-1-1) [29] and experimental values.

The results of the beam K1, with two composite bars, are most concurring with the Japanese guidelines (JSCE 1997) [20] and calculation performed according to the Eurocode Annex [25]. The highest differences between experimental and theoretical values with partial factors were visible for the Canadian [21] and American [23] guidelines.

In the case of beams with higher reinforcement degree, the differences between the standards decreased.

The results of performed tests, regardless of type and degree of reinforcement, were closest to the theoretical values calculated in accordance with Japanese guidelines.

For the calculations of the moments, a simple stress state was assumed for the cross-section, where a force in rebar equals the forces in compression zone. Due to high deformation of the composite reinforcement, the compression zone has low height. In the case of beams with a low degree of reinforcement, the elements exhibit failure through the rapture of rebar. For beams with a high degree of reinforcement, beams fail through the crushing of concrete.

In EN 1992-1 and CNR-DT [24,29] for calculations of the bending moment, the characteristic strength of concrete is taken. In ACI 440 [23], the value of compressive strength is experimentally tested on a cylindrical specimen. The Japanese guidelines (JSCE) [20] take the design strength for the calculations.

Different strengths taken in the calculation can lead to differences in results. For example, in accordance with JSCE, the bearing capacity unintuitively decreases with the increase in the compressive strength used in the calculations. For the purpose of this study, a compressive strength of 55 MPa, reflecting the C55/67 strength class, was used.

Table 4 presents the results of the calculations of load-bearing capacity of studied beams. Three different bending moments were calculated, one based on the experimental results (M_exp_), for a real-life beam. Two were theoretically calculated with (M_rd_) and without (M_rk_) the partial factors. This allowed the determination of the theoretical capacity and design strength of studied beams and the comparison of them to results acquired in the experimental tests. The comparison of the results is presented in Table 5.

The results presented in Table 5 show that the bending capacity obtained in experimental tests of steel-reinforced beam was higher than the theoretical one. In the case of beams reinforced with composite rebar, particularly with a low degree of reinforcement, there is an opposite correlation. The bending capacity obtained in the laboratory tests for beams K1 and K2 was lower than the M_rk_. However, experimental results were higher than the design moments, regardless of the instruction.

The calculations performed for the K1 beam have shown that the lowest capacity reserves were obtained for the EN 1992-1 and JSCE, while high-capacity reserves (>80%) were obtained for CNR and ACI instructions. For the K2 and K3 beams, the lowest capacity reserves were found for the JSCE. The authors think that the experimentally determined capacity should be around 50–75% higher than the designed capacity (M_exp_/M_rd_ > 1.50–1.75), which directly corresponds to the partial factors typically used in calculations. Considering the failure mechanism in beams K1 and K2, where the beams did not achieve the design capacity M_rk_, it seems that the GFRP bar did not achieve their declared strength. This shows that for the safety purposes, the GFRP reinforced elements should be designed to fail through the crushing of concrete.

## 4. Discussion

Design standards for composite rebar typically recommend assuming the failure mechanism through the crushing of concrete [21]. It is possible to achieve failure through rebar, in the case of a low degree of reinforcement [30,31]. However, when insufficient reinforcement is provided, FRP reinforced members can fail due to shear forces [30].

Bentz et al. (2010) [12] studied the effect of reinforcement ratio on large GFRP reinforced concrete members. The study concluded that the behaviour is similar to that of steel-reinforced concrete beams. However, bent FRP reinforcement tends to be significantly weaker at bending due to stress concentrations [22]. The ACI 440.1R-15 [23] requires that FRP strength should be reduced in calculations. Various studies have shown that FRP concrete beams with sufficient shear reinforcement can exhibit failure through crushing of concrete [30,31,32].

None of the beams reinforced with GFRP rebar that were tested in the laboratory have achieved a load-bearing capacity higher than the characteristic capacity calculated using various guidelines without incorporating reducing coefficients.

With the increase in degree of reinforcement, the failure mechanism started to change. For beams with low reinforcement degree (S1, K1), the failure was due to rapture of the rebar, while for beams with four or six bars (K2, K3), the failure was due to crushing of concrete.

Beams used in the study were of smaller size than real-life bended members found in construction. This is why it is important to consider the size effect on the results. According to the Bazant Law [33,34], the cracks in larger elements will appear faster. Similar conclusions were drawn by Karihaloo et al. [35]. The analysis of the crack spacing in GFRP reinforced beams was performed by Syroka et al. [36]. The authors noticed the deflections and cracks width were much higher in beams reinforced with composite rebar. The authors indicated that the failure was caused by shearing of composite bar ribs in a pull-out motion. In the case of beams tested in this study, the shearing of ribs on composite bars was not observed.

According to Di et al. and Baena et al. [37,38], the bonding strength of FRP bars was lower than the steel bars, which could be caused by Poisson effect. In the case of studied reinforcement, due to extensive ribbing, the authors assumed similar resistance to slipping. Thus, the authors assume that the slipping of the bars did not influence the final width and spacing of cracks.

The K1 beam (2#7) had approximately 20% lower load-bearing capacity than the calculated limit values. Considering the expected failure mode, this result shows that the rebar did not achieve assumed tensile strength. The K2 and K3 beams, with higher numbers of reinforcing bars, also did not achieve expected value of bearing capacity. The results were lower than the characteristic capacity calculated using different guidelines.

In every case of beams reinforced with composite rebar, the load-bearing capacity achieved in laboratory tests was in the range of M_rd_–M_rk_. It shows that the failure mode or partial factors incorporated in the calculations should be changed.

Different studies compared various FRP reinforced beams in light of guidelines and standards; some tried to apply the test results to propose new models for calculation of FRP reinforced beams [39,40,41]. Each new study brings us closer to preparing a model with a high degree of compliance.

In bended elements reinforced with the GFRP bars, the rebar often does not achieve the strength declared by the manufacturer. This can happen due to the non-linearity of the element or local stresses in the rebar from concrete. This requires additional studies to determine the cooperation between composite rebar and concrete. Use of partial factors for the reduction of designed strength seems to be the only appropriate move, before we can understand the work of elements reinforced with composite rebar to the full.

## 5. Conclusions

The presented study allowed the following conclusions to be drawn:

1. GFRP reinforced beams during bending tests exhibited cracks of greater depth than those found in steel-reinforced beams. Immediately before failure, the cracks reach up to 90% of the section height and have a much larger width.
2. The use of composite bars, due to their lower modulus of elasticity than steel, increases the deflection of bending elements under the same load, after the section exhibits cracking. The influence of extended deformations on the load-bearing capacity of reinforced elements should be further studied.
3. The serviceability limit state (SLS) plays a much greater role in the case of elements reinforced with composite bars than with steel reinforcement. However, it is necessary to update the limits for elements reinforced with composite rebar. Due to the chemical resistance and lack of corrosion of GFRP bars, the elements will be durable even after extensive cracking. The authors think that the allowed crack width should be increased beyond 0.4 mm.
4. GFRP bars, due to their low Young’s modulus, can be successfully used in elements where the deformability is not important for the structural integrity, e.g., weakly reinforced concrete elements, floors, and thermal insulation connectors.
5. The GFRP reinforcement partial factors for strength reduction recommended by standards and instructions in the calculations of the ultimate limit state reduce the load-bearing capacity of the element so much that the cross-section of the composite reinforcement resembles the cross-section of steel reinforced element. This does not protect against excessive cracks and high deflection. In view of the above, the gain from using GFRP in typical elements should be seen in higher chemical resistance, lower thermal conductivity, and where the reinforcement is only an addition.
6. The anisotropy of the GFRP bars causes susceptibility to local damages, which can result in a lower bearing capacity in bended elements with high deflection and extensive cracks. This might be of great importance in elements with low degree of reinforcement.

Summarising the use of composite reinforcement as an alternative to steel reinforcement in engineering structures requires the development of standardised calculation procedures. The analysis showed that the use of GFRP bars as a replacement for steel bars is possible in demanding environmental conditions. However, excessive deflections and cracks may result in limited application due to overall serviceability requirements of the element.

## Figures and Tables

**Table 1:** The design of the mix used in the study.

Component	Amount
Fine aggregate 0–2 mm	624 kg/m^3^
Coarse aggregate 2–8 mm	1072 kg/m^3^
Cement CEM I 42.5 R	450 kg/m^3^
Fly ash	72 kg/m^3^
Silica fume	38 kg/m^3^
Water	155 kg/m^3^
Superplasticizer SIKA Viscocrete-3	11 kg/m^3^

**Table 2:** Mechanical and physical properties of used reinforcement.

Property	Type of Reinforcement	
	Steel	GFRP
Density (g/cm^3^)	7.85	1.25–2.1
Yield stress (MPa)	500	–
Tensile strength (MPa)	550	>1000
Modulus of elasticity (GPa)	210	35–51
Plastic deformation (%)	0.14–0.125	–
Deformation at failure (%)	6.0–12.0	1.2–3.1

**Table 3:** Maximum crack width and deflection before failure.

	Beam S1	Beam K1	Beam K2	Beam K3
Max. width (mm)	0.4 mm	0.8 mm	0.9 mm	0.8 mm
Deflection	9.1 mm	22.1 mm	15.2 mm	12.3 mm

**Table 4:** Calculation of beams for different standards.

Beam S1 2#8		M_exp_ (kNm)	10.15	
Instruction	EN 1992-1-1	CNR-DT 203/2006	ACI 440.1R-15	JSCE 1997
Characteristic M_rk_ (kNm)	8.82	-	-	-
Design M_rd_ (kNm)	7.61	-	-	-
Failure mechanism	bars			
Partial factors	γ_s_ = 1.15; γ_c_ = 1.50			
Beam K1 2#7		M_exp_ (kNm)	10.73	
Instruction	EN 1992-1-1	CNR-DT 203/2006	ACI 440.1R-15	JSCE 1997
Characteristic M_rk_ (kNm)	13.35	13.35	12.43	13.35
Design M_rd_ (kNm)	10.21	5.78	5.82	10.46
Failure mechanism	bars	bars	bars	bars
Partial factors	γ_f_ = 1.30; γ_c_ = 1.50	γ_m_ = 1.50; η_a_ = 0.8; η_l_ = 0.8;	β_1_ = 0.654; C_E.G_ = 0.8; Φ = 0.55	γ_mf_ = 1.15; γ_c_ = 1.50
Beam K2 4#7		M_exp_ (kNm)	16.25	
Instruction	EN 1992-1-1	CNR-DT 203/2006	ACI 440.1R-15	JSCE 1997
Characteristic M_rk_ (kNm)	16.74	16.74	16.79	21.82
Design M_rd_ (kNm)	11.16	11.16	10.92	13.86
Failure mechanism	concrete	concrete	concrete	concrete
Partial factors	γ_f_ = 1.30; γ_c_ = 1.50	γ_m_ = 1.50; η_a_ = 0.8; η_l_ = 0.8;	β_1_ = 0.654; C_E.G_ = 0.8; Φ = 0.65	γ_mf_ = 1.15; γ_c_ = 1.50
Beam K3 6#7		M_exp_ (kNm)	19.15	
Instruction	EN 1992-1-1	CNR-DT 203/2006	ACI 440.1R-15	JSCE 1997
Characteristic M_rk_ (kNm)	16.74	16.74	19.87	25.82
Design M_rd_ (kNm)	11.16	11.16	12.92	16.17
Failure mechanism	concrete	concrete	concrete	concrete
Partial factors	γ_f_ = 1.30; γ_c_ = 1.50	γ_m_ = 1.50; η_a_ = 0.8; η_l_ = 0.8;	β_1_ = 0.654; C_E.G_ = 0.8; Φ = 0.65	γ_mf_ = 1.15; γ_c_ = 1.50

γ_f_; C_E,G_; γ_m_; γ_mf_—partial factors in the ULS; γ_c_—partial factor for concrete in ULS; η_a_—partial factor for exposition; η_l_—partial factor for long-term loads; Φ—partial factor for degree of composite reinforcement; β_1_—partial factor for concrete strength.

**Table 5:** Calculation of beams load capacity ratios for different standards.

	Beam S1 2#8			
M_exp_/M_rk_	1.15			
M_exp_/M_rd_	1.33			
	Beam K1 2#7			
Instruction	EN 1992-1-1	CNR-DT 203/2006	ACI 440.1R-15	JSCE 1997
M_exp_/M_rk_	0.80	0.80	0.86	0.80
M_exp_/M_rd_	1.05	1.86	1.84	1.03
	Beam K2 4#7			
Instruction	EN 1992-1-1	CNR-DT 203/2006	ACI 440.1R-15	JSCE 1997
M_exp_/M_rk_	0.97	0.97	0.97	0.74
M_exp_/M_rd_	1.46	1.46	1.49	1.17
	Beam K3 6#7			
Instruction	EN 1992-1-1	CNR-DT 203/2006	ACI 440.1R-15	JSCE 1997
M_exp_/M_rk_	1.14	1.14	0.96	0.74
M_exp_/M_rd_	1.72	1.72	1.48	1.18

## Data Availability

Data available on request due to file type and size.
